# The effects of consuming a Mediterranean style diet on associated COVID-19 severity biomarkers in obese/overweight adults: A systematic review

**DOI:** 10.1177/02601060221127853

**Published:** 2022-09-21

**Authors:** Ella Moore, Abdulmannan Fadel, Katie E. Lane

**Affiliations:** Research Institute for Sport and Exercise Science, School of Sport and Exercise Sciences, 4589 Liverpool John Moores University, Liverpool, UK

**Keywords:** COVID-19, non-communicable disease, BMI, biomarkers, hypocaloric diet, Mediterranean diet

## Abstract

**Background:** COVID-19 severity is strongly associated with high Body Mass Index (BMI) (≥25kg/m^2^) amongst adults and elevated inflammatory markers have enabled prediction of disease progression. The composition of a Mediterranean diet provides favourable outcomes on weight reduction and inflammatory markers. **Aim:** This systematic review aimed to investigate the effects of consuming a Mediterranean diet on BMI and inflammatory markers of obese/overweight adults (≥18 years) at risk of developing severe COVID-19 outcomes. **Methods:** PubMed Central, Cochrane Library and MEDLINE databases were searched to identify randomised controlled trials published between January 2010 to August 2021 evaluating the impact of Mediterranean diet on BMI and inflammatory markers in overweight/obese adults. The review followed the PRISMA checklist, used Cochrane Collaboration search strategies, and is PROSPERO registered (CRD42021277070). Two authors independently screened and evaluated studies for methodological quality. Papers were extracted and included based eligibility, despite risk of bias scores. **Results:** Of 65 extracted records, six studies met the eligibility criteria and were included. Reductions in BMI, TNF-α, IL-6 and hs-CRP were reported amongst most findings, the majority of which were significant. **Conclusion:** The main findings indicate a hypocaloric, fibre dense Mediterranean diet is a short-term (<4 months) mitigation strategy to significantly reduce BMI and inflammatory markers amongst overweight/obese adults at risk of developing severe COVID-19 outcomes. Further research is now needed to examine the role of Mediterranean diet in COVID-19 prevalence, severity, morbidity and mortality.

## Introduction

On 12th January 2020 a novel coronavirus was identified in Wuhan City, Hubei Province, China. The virus is referred to as SARS-CoV-2, with the associated disease as COVID-19 (World Health Organisation, 2021). COVID-19 is an infectious disease, spread primarily through droplets of saliva or discharge from the nose when an infected person coughs or sneezes. The most common symptoms are fever, dry cough, loss of taste and smell and fatigue (World Health Organisation, 2021). COVID-19 has spread to every inhabited continent worldwide, accumulating so far to over 596,873,121 confirmed cases globally, with more than 6,459,684 mortalities with ongoing escalation in cases confirmed and related mortalities (World Health Organisation, 2022b; Zabetakis et al., 2020).

It is understood that those who are at a greater risk of developing more severe outcomes from COVID-19 include older individuals, aged 65 years and over (Luo et al., 2020; Yanez et al., 2020), as well as those who are immunocompromised (Fung and Babik, 2020) and with underlying medical conditions, such as cardiovascular disease (CVD) (Bae et al., 2021; Bansal, 2020), type 1 and type 2 diabetes (Barron et al., 2020; Dennis et al., 2021), cancer (Al-Quteimat and Amer, 2020; Carreira et al., 2020) and obesity (de Siqueira et al., 2020; Du et al., 2021; Huang et al., 2020). Obesity is the abnormal or excessive fat accumulation that may impair health (World Health Organisation, 2022a) therefore obesity may and usually does produce many metabolic disturbances, whereby the severity of COVID-19 is further heightened in parallel with Body Mass Index (BMI) (Iannelli et al., 2020). These co-existing factors have demonstrated an increase in the likelihood of severe illness, particularly in those with a BMI of >30 kg/m^2^ (de Siqueira et al., 2020; Du et al., 2021; Huang et al., 2020), potentially resulting in hospitalisation, medical complications, intensive care unit (ICU) admission and an almost 3-fold risk for COVID-19 related mortality (Du et al., 2021; Singh et al., 2020). However, even those who are overweight, with a BMI of ≥25 kg/m^2^, are exposed to an increased risk of serious illness and death from COVID-19, as well as a >20% higher risk of contracting COVID-19 than individuals with adequate body weight (Hamer et al., 2020; Jung et al., 2021).

Continued follow up of patients recovering from COVID-19 infection shows symptoms can persist in many people during the following weeks and months after the initial infection (Yong, 2021). Long COVID is common, affecting up to one in five people testing positive for COVID-19 and denotes the persistence or relapse of symptoms irrespective of virus status (Shah et al., 2021). High BMI and pre-infection elevated inflammatory markers are thought be risk factors for long COVID (Vimercati et al., 2021) leading to unresolved inflammation from multiple sources (Del Valle et al., 2020; Yong, 2021).

There are an array of health benefits linked to consumption of a Mediterranean (MD), typically high in plant-based foods (fruit, vegetables, nuts and cereals), olive oil, moderate intakes of fish and poultry and low intakes of dairy products (yoghurt and cheese), red meat, processed meats and sweets (World Health Organisation, 2018). High adherence to a MD has been associated with reduced mortality from coronary heart disease (Trichopoulou et al., 2005), lower risk of incident coronary heart disease and stroke (Fung et al., 2009), reduced age-related weight gain (Beunza et al., 2010), lower abdominal adiposity (Romaguera et al., 2009), reduced risk of diabetes (Martínez-González et al., 2008) and a reduced risk of developing mild cognitive impairment (Scarmeas et al., 2009). It is believed the health benefits of the MD are from multiple interactions, whereby the intake of various macro- and micronutrients accompanied with many biologically active compounds lead to a favourable effect on the mitigation or prevention of various diseases (Lăcătușu et al., 2019; Romaguera et al., 2009; Trichopoulou et al., 2005). The interaction between nutrient and food variation presents positive enhancements regarding the prevention of health concerns, even when consumed in the absence of calorie restriction (Salas-Salvadó et al., 2011).

Multiple MD studies have shown protective effects on obesity and the prevention of weight gain (Agnoli et al., 2018; Mendez et al., 2006; Schröder, 2007) as well as significant alterations in inflammatory markers seen elevated in those with severe cases of COVID-19, such as interleukin 6 (Medina-Remón et al., 2017; Schwingshackl and Hoffmann, 2014) and high-sensitive C reactive protein (Schwingshackl and Hoffmann, 2014). It seems adopting this diet could mitigate the likelihood of developing more serious COVID-19 outcomes, through weight reduction, immune system support and anti-inflammatory properties.

Lifestyle approaches to mitigate unfavourable outcomes of COVID-19 should create the foundation for the current study, as it is evident that a healthy lifestyle and maintaining an adequate body weight is essential in preventing and reducing poor COVID-19 outcomes (Soeroto et al., 2020). In line with this, The World Health Organisation has announced new dietary guidelines to outline the importance of a balanced diet to maintain a strong immune system to reduce chronic diseases and infections (World Health Organisation, 2019). Similarly to the traditional MD, the new proposed dietary guidelines advise on consuming four servings of fruit per day, five servings of vegetables per day, alongside 180g of wholegrain cereals and a 160g combination of meats and beans (Jayawardena and Misra, 2020; World Health Organisation, 2020).

### Systematic review rationale

To date, no systematic review has evaluated the effects of a MD on BMI and inflammatory markers for overweight and/or obese adults and applied this evidence as a potential mitigation strategy for COVID-19 severity. This review aims to systematically identify and evaluate research in attempt to answer the following question: What are the effects of consuming a Mediterranean style diet on the BMI and inflammatory markers of obese/overweight adults? The research objectives were as follows: (1) Evaluate whether there is a significant difference in BMI for obese and/or overweight adults who consume a Mediterranean style diet; (2) Identify the effect of consuming a Mediterranean style diet on inflammatory markers in overweight and/or obese adults; (3) Explore the potential health outcomes of consuming a Mediterranean style diet by obese and/or overweight adults in light of the COVID-19 pandemic.

## Methods

### Review design

The current systematic review follows the Preferred Reporting Items for Systematic reviews and Meta-Analyses (PRISMA) guidelines (Moher et al., 2009), additional nutrition-based systematic review guidance (Kelley and Kelley, 2019) and is registered in the International Prospective Register of Systematic Reviews (PROSPERO CRD42021277070 (Moore et al., 2021)). Peer-reviewed literature was searched to accumulate studies in overweight and/or obese adults (≥18 years) where a MD was used as the intervention (high intake of plant-based foods, olive oil, moderate intake of fish and poultry and low intake of dairy products, red meat, processed meats and sweets). The outcome measures of interest included baseline and endpoint measures of BMI and inflammatory markers. Evidence provided by this systematic review, combined with the knowledge and expertise of healthcare professionals, findings could be utilised in future decision making and intervention delivery as we progress through the COVID-19 pandemic.

### Search strategy

A search of PubMed Central (PMC), Cochrane Library and MEDLINE (EBSCO) was conducted using PRISMA guidelines (Esposito et al., 2004; Mancini et al., 2016). The search strategy was created and implemented from the population, exposure, outcome (PEO) framework, detailed in Table 1, to extract the most up to date studies between 01/01/2010 to 01/08/2021. The main search terms included “overweight”, “obese”, “adult”, “Mediterranean style diet”, “body mass index” and “inflammatory”. Full search strategies used for each electronic database are presented in Table 2, with the implementation of Boolean operators (Boland et al., 2017). Identified studies were exported into “Endnote” bibliography software (version X9, Thomson Reuters, New York) for collation and refinement. To further minimise effects of publication bias, a snowball method, characterised by manual checking of references from retrieved articles, was applied to ensure complete collection. Publication alerts were set to identify any studies published after the date of the literature search.

**Table 1.** Formation of full search strategy using PEO framework for database searches to retrieve relevant studies.

Search	Search strategy
**P**opulation (S1)	Overweight OR Over-weight OR over weight OR obes* OR fat **[title/abstract]**
(S2)	Adult OR adults OR grown*up OR men OR man OR woman OR women **[title/abstract]**
**E**xposure (S3)	“Mediterranean style diet” OR “Mediterranean diet” OR “Med Diet” OR meddiet OR “Mediterranean dietary pattern” **[title/abstract]**
**O**utcome (S4)	“Body Mass Index” or BMI **[title/abstract]**
(S5)	Inflammatory OR inflammation **[title/abstract]**
(S6)	(S1) AND (S2) AND (S3) AND (S4) AND (S5)

**Table 2.** Electronic database search strategy used for each database (PubMed Central, Cochrane Library and MEDLINE).

Database/Repository	Terms Searched	Additional Qualifiers	Papers Retrieved	
			Initial search	Revised search
PubMed Central (PMC)	(((((((Overweight[Abstract] OR Over-weight[Abstract] OR over weight[Abstract] OR obes*[Abstract] OR fat[Abstract])) OR (Overweight[Title] OR Over-weight[Title] OR over weight[Title] OR obes*[Title] OR fat[Title]))) AND (((Adult[Abstract] OR adults[Abstract] OR grown*up[Abstract] OR men[Abstract] OR man[Abstract] OR woman[Abstract] OR women[Abstract])) OR (Adult[Title] OR adults[Title] OR grown*up[Title] OR men[Title] OR man[Title] OR woman[Title] OR women[Title]))) AND (((“Mediterranean style diet”[Abstract] OR “Mediterranean diet”[Abstract] OR “Med Diet”[Abstract] OR meddiet[Abstract] OR “Mediterranean dietary pattern"[Abstract])) OR (“Mediterranean style diet”[Title] OR “Mediterranean diet”[Title] OR “Med Diet”[Title] OR meddiet[Title] OR “Mediterranean dietary pattern"[Title]))) AND (((“Body Mass Index”[Abstract] OR BMI[Abstract])) OR (“Body Mass Index”[Title] OR BMI[Title]))) AND (((Inflammatory[Abstract] OR inflammation[Abstract])) OR (Inflammatory[Title] OR inflammation[Title]))	Filters applied; Peer-reviewed Articles published from 01/01/2010 to 31/11/2020. Articles published in the English language	14	14
Cochrane Library	(Overweight OR Over-weight OR over weight OR obes* OR fat) AND (Adult OR adults OR grown*up OR men OR man OR woman OR women) AND (“Mediterranean style diet” OR “Mediterranean diet” OR “Med Diet” OR meddiet OR “Mediterranean dietary pattern”) AND (“Body Mass Index” or BMI) AND (Inflammatory OR inflammation)		31	27
MEDLINE (EBSCO)	AB (Overweight OR Over-weight OR over weight OR obes* OR fat) OR TI (Overweight OR Over-weight OR over weight OR obes* OR fat) AND AB (Adult OR adults OR grown*up OR men OR man OR woman OR women) OR TI (Adult OR adults OR grown*up OR men OR man OR woman OR women) AND AB (“Mediterranean style diet” OR “Mediterranean diet” OR “Med Diet” OR meddiet OR “Mediterranean dietary pattern”) OR TI (“Mediterranean style diet” OR “Mediterranean diet” OR “Med Diet” OR meddiet OR “Mediterranean dietary pattern”) AND AB (“Body Mass Index” or BMI) OR TI (“Body Mass Index” or BMI) AND AB (Inflammatory OR inflammation) OR TI (Inflammatory OR inflammation)		27	24
**Total**			72	65
**Total after refining of journal results**				6

### Inclusion/exclusion criteria

Once duplicate papers were removed, study screening began by reviewing titles and abstracts of retrieved articles using the search strategies in Table 2. Studies that met the eligibility criteria underwent a full-text review for inclusion or exclusion. The following inclusion criteria was applied: (1) studies must be peer-reviewed articles with full-text accessibility in English (2) participants must be aged 18 years and over (3) participants consuming a MD intervention must have a mean baseline BMI of ≥25kg/m^2^ (4) use of a MD as the intervention in randomised controlled trials (RCT) and case control studies, (5) Studies must use appropriate quantitative measures of BMI and inflammatory markers to compare pre- and post-intervention. Studies were excluded on the following criteria: (1) studies did not include baseline and endpoint quantitative measures of BMI and/or inflammatory markers (2) additional non-dietary interventions were introduced (e.g., exercise/activity programmes) (3) studies that were still active. The inclusion criteria contained no restrictions on the following (1) type of MD intervention (e.g., hypocaloric, enriched with Extra Virgin Olive Oil) (2) intervention/follow up period (3) participant sex (4) participant ethnicity (5) sample size (6) study location/country. All included participants remained in absence of serious, non-obesity related illnesses. To capture as many studies as possible, the following participant factors remained eligible: (1) post-partum breastfeeding women (2) participants with a CVD risk profile (3) medicated coronary artery disease secondary prevention patients (4) participants with metabolic syndrome.

### Data extraction and synthesis

Appropriate study characteristics were extracted into Table 3. To minimise potential bias during the selection procedure, articles retrieved were independently read by 2 reviewers (EM and AF). A third reviewer (KEL) then made a consensus decision for inclusion. The articles were added to an independent Endnote database and grouped in accordance with the inclusion criteria. These data items included: (i) general characteristics of the study (first authors name, publication year, design and country), (ii) participant demographics (age, sex, mean baseline BMI), (iii) study characteristics (sample size, follow up period, study arms, eligibility criteria and object of study). Data regarding outcome measurements were extracted, as well as their significance value, including baseline and endpoint BMI measurements and inflammatory marker figures, including C-reactive Protein (CRP), High-Sensitivity C-Reactive Protein (hs-CRP), Tumour Necrosis Factor-α (TNF-α), Interleukins (IL-1ra, IL-4, IL-6, IL-8, IL-10, IL-12 and IL-17), Plasminogen Activator Inhibitor-1 (PAI-1), Malondialdehyde (MDA), Interferon-Gamma (IFN-γ), Monocyte Chemoattractant Protein-1 (MCP-1), Macrophage Inflammatory Protein (MIP-1β), Vascular Endothelial Growth Factor (VEGF) and Interferon-γ–induced protein (IP-10) (Table 4). Data regarding dietary interventions and dietary adherence is included in Table 5. A meta-analysis was not deemed appropriate due to variability in components of the MD. Therefore, the entire body of studies was summarised descriptively and a qualitative synthesis of the studies included was performed.

**Table 3.** Study characteristics.

Author, Year & Country	Type of study	Study aim	Age (y), number of participants	Eligibility criteria	Arms	Follow up	Main findings
Stendell-Hollis et al. (2013) USA	Randomised, controlled dietary intervention trial	To assess the effects of a MED diet and the United States Department of Agriculture's MyPyramid diet for Pregnancy and Lactation on body weight, adiposity, and biomarkers of inflammation, tumour necrosis factor-α (TNF-α) and IL-6, in overweight, postpartum, breastfeeding women.	29.7±4.6, N=129 females	Inclusion: aged 18-40, plan to breastfeed ≥6 months, no hormonal contraceptive use, breastfeeding ≥3 per day, no food allergy. Exclusion: used tobacco, personal/family history of food allergies	Mediterranean-style (MED) diet	4 months	Both diet groups demonstrated significant (p<0.001) reductions in body weight (−2.3±3.4 kg and −3.1±3.4 kg for the MED and comparison diets, respectively). A significant decrease in TNF-α but not IL-6 was also demonstrated in both diet groups, with no significant between-group difference.
					United States Department of Agriculture's (USDA) MyPyramid diet for Pregnancy and Lactation		
Marques-Rocha et al. (2016) Spain	Controlled intervention study	To evaluate the influence of a dietary strategy for weight loss on the expression of inflammation-related microRNAS (miRNAs) and genes in white blood cells (WBC) from individuals with metabolic syndrome (MetS).	48.84 ±10.02, N=40 (M=20, F=20)	Inclusion: aged 35-65, central obesity, plus any two of the following four factors: raised triglycerides, reduced HDL - cholesterol, raised blood pressure, raised fasting plasma glucose. Excluded: Psychiatric disturbances, eating disorders, chronic diseases related to the metabolism of nutrients, major body weight changes in the previous 3 months, and difficulties in changing food habits	RESMENA Diet	8 weeks	Participants demonstrated a significant decrease in BMI (P<0.01) alongside significant reductions in MDA (P<0.01) and PAI-1 (P<0.01). Non-significant increases were also reported in CRP, IL-6 and TNF-a.
Sofi et al. (2018) Italy	Randomised, open, crossover controlled trial	To compare, in a population of omnivorous overweight individuals living in a low-risk (for cardiovascular disease) European country, the effects of a 3-month period on a low-calorie lacto-ovo vegetarian diet compared with a low-calorie Mediterranean diet (MD) on several markers of cardiovascular disease risk.	50 (21–75), N=118 LCMD (N=58, F=43, M=15) LCVD (N=60, F=49, M=11) N=107 included in analysis (LCMD=103, LCVD=104).	Included: BMI ≥25 kg/m², simultaneous presence of ≥1 of the following criteria:15 total cholesterol levels >190 mg/dL, low-density lipoprotein (LDL) cholesterol levels >115 mg/dL, triglyceride levels >150 mg/dL, and glucose levels >110 but <126 mg/dL Excluded: taking medications, serious illness or an unstable condition, pregnant or nursing, participating or had participated in a weight loss treatment program in the last 6 months, following or had followed a food profile that excluded meat, poultry, or fish in the last 6 months.	Low-Calorie Vegetarian diet (LCVD)	3 months	Both groups presented significant decreases in BMI (P<0.05). Participants in the MD group demonstrated significant reductions in IL-1ra (P<0.05), IL-12 (P<0.05), IL-17 (P<0.05), MCP-1 ((P<0.05) and VEGF (P<0.05), alongside significant increases in IL-4 (P<0.05). Non-significant reductions in IL-6, IL-8, IL-10, TNF-α and IP-10 were also reported in the MD group, alongside non-significant increases in IFN-γ.
					Hypocaloric Mediterranean Diet (MD)		
Perticone et al. (2019) Italy	Controlled, randomised, open design	To investigate if weight loss obtained through VLCKD is associated with an increase in serum 25(OH)D concentration amongst overweight adults.	46.75±11.05, N=56 SHMD (N=28, M=18, F=10) VLCKD (N=28, M=14, F=14)	Included: aged > 18 years and BMI > 30 kg/m² Excluded: pregnancy, breastfeeding, type-1 diabetes mellitus, heart failure, history or clinical evidence of angina, myocardial infarction, valvular heart disease, neoplastic disease, estimated glomerular filtration rate (eGFR) < 60 mL/min/1.73 m2, liver dysfunction, use of medications able to interfere with glucose and/or vitamin D metabolism, thyroid disorders, and history or clinical evidence of psychiatric disorders.	Very low-calorie (<800 kcal per day) ketogenic diet (VLCKD)	12 months	Non-significant reductions in BMI were reported amongst both intervention groups. Significant reductions were reported in hs-CRP amongst participants in the SHMD group (P=0.044) and the VLCKD group (P<0.0001).
					Standard hypocaloric Mediterranean diet (SHMD)		
Luisi et al. (2019) Italy	Cohort case control	To study, if and how, some parameters of inflammation and oxidative stress and GM’s LAB number copies, change after 3 months of MD rich in High Quality-Extra Virgin Olive Oil (40g/day) in a cohort of overweight/obese subjects in comparison with normal weight controls.	Cases: 52.1± 13.04 (N=18, M=11, F=7) Controls: 41.4 ± 14.42 (N=18, M=6, F=12)	Included: BMI ≥18.5 kg/m². Excluded: Subjects suffering of eating disorders and in recent (1 month) or ongoing antibiotic therapy.	Overweight/obese subjects (cases, BMI ≥25 kg/m²) fed with low calorie Mediterranean Diet	3 months	Significant reductions in BMI were reported amongst overweight/obese cases (P<0.01). Non-significant BMI reductions were also reported amongst controls. Amongst overweight/obese cases, significant reductions were reported in TNF-α (P < 0.001) and IL-6 (p < 0.001), alongside a significant increase in IL-10 (p< 0.001).
					Normal weight controls (BMI 18.5–24.9 kg/m²) fed with Mediterranean Diet		
Thomazella et al. (2011) Brazil	Prospective controlled clinical trial	To compare, in medicated patients with coronary artery disease, the effects of aggressive treatment with the Mediterranean diet (MD) to those with the Therapeutic Lifestyle Changes Diet (TLCD), with a focus on endothelial function, inflammation, and oxidative stress.	MD= 55.0 ± 4.6 (N=21, M=21) TLCD=54.6 ± 5.0 (N=19, M=19)	Included: ≥1 coronary event occurring <24 and >4 months before enrollment, clinical stability and absence of secondary events, BMI 18.5 to 30.0 kg/m², nonsmoker or ex-smoker for >1 year, and fasting blood glucose <110 mg/dl. Excluded: history of diabetes, chronic illnesses, or food allergy; serum low-density lipoprotein (LDL) >190 mg/dl; serum triglycerides >310 mg/dl; drug or alcohol addiction; and any condition that might impair participation in the study.	Mediterranean diet (MD)	3 months	Signification reductions in BMI were reported amongst both intervention groups (< 0.001). A non-significant increase in hs-CRP was reported in the TLCD group, whereas a non-significant decrease was reported in the MD group.
					low-fat Therapeutic Lifestyle Changes Diet (TLCD)		

HDL, high density lipoprotein.

**Table 4.** Changes in body mass index (BMI) and inflammatory markers from baseline to endpoint.

Study	Follow up period	Body Mass Index			Inflammatory markers			
		Baseline (kg/m^2^)	Endpoint(kg/m^2^)	P value	Inflammatory marker	Baseline	Endpoint	P value
**Stendell-Hollis et al. (2013)**								
Mediterranean-style (MED) diet	4 months	27.1±5.29	26.2±5.58 (−0.85±1.24)***	<0.001	IL-6, pg/mL TNF-α, pg/mL	1.977 (0.68-3.27) 4.189 (1.30-7.08)	1.585 (0.84-2.33) 3.301 (1.10-5.50)*	Not significant 0.021
United States Department of Agriculture's (USDA) MyPyramid diet for Pregnancy and Lactation		26.7±4.82	25.6±5.24 (−1.13±1.22)***	<0.001	IL-6, pg/mL TNF-α, pg/mL	0.916 (0.71-1.13) 2.475 (1.48-3.47)	0.888 (0.66-1.11) 1.940 (1.18-2.70)***	Not significant <0.001
**Marques-Rocha et al. (2016)**								
RESMENA Diet	8 weeks	35.4±4.4	32.8±4.2**	<0.01	MDA, mM CRP, mg/L IL-6, pg/mL PAI-1, pg/mL TNF-a, pg/mL	0.84±0.36 3.30±3.45 2.63±1.73 157±127 0.69±0.50	0.74±0.28** 3.53±5.75 2.76±1.58 144±152** 0.88±0.94	<0.01 0.71 0.53 <0.01 0.99
**Sofi et al. (2018)**								
Low-Calorie Vegetarian diet	3 months	30.1±4.7	−0.67 kg/m^2^*	<0.05	Interleukin-1ra, pg/mL Interleukin-4, pg/mL Interleukin-6, pg/mL Interleukin-8, pg/mL Interleukin-10, pg/mL Interleukin-12, pg/mL Interleukin-17, pg/mL MCP-1, pg/mL MIP-1β, pg/mL VEGF, pg/mL TNF-α, pg/mL IP-10, pg/mL IFN-γ, pg/mL	11.62 (9.82–13.76) 0.07 (0.05–0.09) 0.74 (0.60–0.92) 3.39 (2.72–4.22) 1.71 (1.32–2.21) 15.46 (13.40–17.85) 3.70 (2.82–4.86) 21.24 (18.90–23.88) 48.91 (43.90–54.43) 39.88 (33.72–47.18) 3.05 (2.23–4.17) 479.62 (435.72–527.95) 3.58 (2.87–4.46)	10.33 (8.76–12.18) 0.12 (0.09–0.16)* 0.81 (0.66–1.00) 2.86 (2.27–3.61) 1.83 (1.41–2.39) 15.43 (13.40–17.74) 5.09 (4.14–6.26)* Ω 19.13 (17.03–21.50)* 45.11 (41.26–49.25) 35.30 (29.99–41.55)* 3.50 (2.92–4.18) 434.41 (393.07–v480.10)* 2.66 (2.06–3.43)*	Not significant P<0.05 Not significant Not significant Not significant Not significant P<0.05 P<0.05 Not significant P<0.05 Not significant P<0.05 P<0.05
Low-Calorie Mediterranean Diet		31.1±5.1	−0.64 kg/m^2^*	<0.05	Interleukin-1ra, pg/mL Interleukin-4, pg/mL Interleukin-6, pg/mL Interleukin-8, pg/mL Interleukin-10, pg/mL Interleukin-12, pg/mL Interleukin-17, pg/mL MCP-1, pg/mL MIP-1β, pg/mL VEGF, pg/mL TNF-α, pg/mL IP-10, pg/mL IFN-γ, pg/mL	13.45 (11.43–15.82) 0.07 (0.05–0.09) 0.84 (0.68–1.04) 3.35 (2.69–4.18) 1.81 (1.37–2.37) 16.48 (14.11–19.26) 5.51 (4.54–6.69) 22.76 (20.05–25.87) 52.40 (47.66–57.57) 42.86 (35.80–51.32) 3.20 (2.53–4.04) 475.33 (427.95–527.95) 2.53 (1.93–3.30)	10.70 (9.23–12.39)* 0.12 (0.09–0.16)* 0.75 (0.63–0.90) 3.01 (2.42–3.75) 1.50 (1.14–1.95) 14.35 (12.45–16.59)* 3.51 (2.68–4.61)* Ω 17.98 (16.17–19.97)* 45.47 (41.06–50.40)* 36.16 (30.51–42.91)* 2.86 (2.12–3.87) 447.20 (407.48–490.78) 3.22 (2.58–4.00)	P<0.05 P<0.05 Not significant Not significant Not significant P<0.05 P<0.05 P<0.05 P<0.05 P<0.05 Not significant Not significant Not significant
**Perticone et al. (2019)**								
Very low-calorie (<800 kcal per day) ketogenic diet (VLCKD)	12 months	40.5 ± 10.8	33.3 ± 9.72	0.212	hs-CRP, mg/L	4.5 ± 2.6	1.8 ± 0.8****	<0.0001
Standard hypocaloric Mediterranean diet (SHMD) (500 kcal/day caloric deficit)		38.8 ± 4.5	36.1 ± 5.7	0.321	hs-CRP, mg/L	5.6 ± 4.3	3.7 ± 1.2*	0.044
**Luisi et al. (2019)**								
Normal weight controls (BMI 18.5–24.9 kg/m^2^) fed with typical Mediterranean Diet	**3** months	21.6 ± 0.6	21.7 ± 0.6	Not significant	TNF-α, pg/mL IL-6, ng/mL IL-10, pg/mL	1.6 4 7	1.4 *** 3.2 *** 7.5	p < 0.001 p < 0.001 Not significant
Overweight/obese subjects (cases, BMI ≥25 kg/m^2^) fed with low-calorie Mediterranean Diet		30.2 ± 1.0	28.8 ± 0.9** Ω	P < 0.01	TNF-α, pg/mL IL-6, ng/mL IL-10, pg/mL	1.5 4.67 5.5	1.1 *** 3.87 *** 7.5 *** Ϯ	p < 0.001 p < 0.001 p< 0.001
**Thomazella et al. (2011)**								
Mediterranean diet (MD)	**3** months	26.5 ± 1.9	25.9 ± 1.8***	< 0.001	hs-CRP, mg/L	1.65 ± 1.50	1.07 ± 0.93	Not significant
Therapeutic Lifestyle Changes Diet (TLCD)		26.3 ± 2.5	25.7 ± 2.4***	< 0.001	hs-CRP, mg/L	1.38 ± 1.07	2.07 ± 2.99	Not significant

**** P < 0.0001, *** P < 0.001, ** P < 0.01, *P < 0.05 for within-group changes from baseline

Ω - significant difference between groups (P < 0.05), Ϫ - significant difference between groups (P < 0.01), Ϯ – significant difference between groups (p < 0.001)

**Table 5.** Summary of diet interventions and measures of dietary adherence for included studies.

Reference	Mediterranean diet intervention	Control intervention or assessment	Mediterranean diet adherence outcome
Stendell-Hollis et al. (2013)	Mediterranean-style (MED) diet Dietary intake measure: Validated Food Frequency Questionnaire (FFQ) . MED scores were calculated with results ranging from 0–9, with 9 indicating the best adherence Diet components: ≥6 servings of whole grains per day, ≥7 servings/day of fruits and vegetables, legumes, nuts, walnuts (28 g/day), ≥2 servings of fish per week, poultry, 1–2 tablespoons/day of olive oil (refined or virgin), low-fat dairy products, limiting the intake of whole fat dairy products, red meats, processed foods, desserts, and sources of fat other than olive oil. Participants were instructed to consume the study-provided prenatal vitamin daily.	USDA MyPyramid for Pregnancy and Breastfeeding Dietary intake measure: Validated Food Frequency Questionnaire (FFQ) MED scores were calculated with results ranging from 0–9, with 9 indicating the best adherence. Diet components: General nutrition education guidelines based on the USDA MyPyramid diet for Pregnancy and Breastfeeding, emphasising healthy eating choices. Intake of nuts, the use of olive oil, and an increase in fruits and vegetables were deemphasized in order to differentiate the diet from the MED diet Participants were instructed to consume the study-provided prenatal vitamin daily.	MED score Baseline score: 4.08 Endpoint score: 4.76
Marques-Rocha et al. (2016)	RESMENA diet Dietary intake measure: Semiquantitative 136-item food frequency questionnaire and The Healthy Eating Index (HEI). The final value was classified into five categories: >80 points indicates “excellent diet”; 71 to 80 points, a “very good diet”; 61 to 70 points, a “good diet”; 51 to 60, an “acceptable diet”; and 0 to 50 points, an “inadequate diet.” Diet components: Weight loss strategy based on the Mediterranean dietary pattern (the RESMENA [reduction of metabolic syndrome in Navarra, Spain] diet). Subjects were advised to consume 7 meals per day, including breakfast, lunch, dinner, two snacks in the morning, and two more snacks in the afternoon. Energy restriction of 30% applied to the total energy requirements of each patient (resting metabolic rate was calculated using the Harris–Benedict equation). Moderately high protein intake (24.6% ± 2.8%) Increased total antioxidant capacity (TAC) than the usual recommendations. Cholesterol content <300 mg Low glycemic index and glycemic load (GL) carbohydrate meals	N/A	Healthy Eating Index (U) Baseline score: 55.9 ± 11.8 Endpoint score: 71.4 ± 10.2
Sofi et al. (2018)	Low-calorie Mediterranean diet Dietary intake measure: Mediterranean Diet adherence score. Participants considered adherent if reported ≥10 points in a scale ranging from 0 to 18. Diet components: Consumption of all the food groups, including meat and meat products, poultry, and fish. 50% to 55% of energy from carbohydrate 25% to 30% of energy derived from total fat (≤7% of energy from saturated fat, <200 mg/d of cholesterol) 15% to 20% of energy derived from protein	Low-calorie vegetarian diet Dietary intake measure: 24-hour diet recall and a food frequency questionnaire Diet components: Abstinence from the consumption of meat and meat products, poultry, fish, and seafood, and the flesh of any other animal. It included eggs and dairy products, as well as all the other food groups. 50% to 55% of energy from carbohydrate 25% to 30% of energy derived from total fat (≤7% of energy from saturated fat, <200 mg/d of cholesterol) 15% to 20% of energy derived from protein	LCMD: Total of 9 withdrew, 7 of which due to lack of adherence. LCVD: total of 9 withdrew, 8 of which due to lack of adherence
Perticone et al. (2019)	Standard Hypocaloric Mediterranean Diet Dietary intake measure: Self-reports and food records Diet components: Patients in this group followed a balanced diet allowing the use of whole grain pasta, bread, rice, meat, fish, eggs, and vegetables in different combinations, as prescribed by an experienced dietitian. Caloric deficit: 500 kcal/day (based on basal metabolic rate) 55%–60% of energy derived from carbohydrates 10%–15% of energy derived from proteins 25%–30% of energy derived from lipids	Very Low-Calorie Ketogenic Diet Dietary intake measure: Self-reports and food records Diet components: All nutritional requirements were met using five to six formulated meals a day. Energy intake: of 600 kcal per day 50%–60% of energy intake derived from proteins 20%–30% of energy derived from lipids 20% of energy derived from carbohydrates	(SHMD) Four patients were lost to follow-up, and only 55% of the remaining patients achieved an acceptable degree of adherence. (VLCKD) 95% of patients showed a good compliance to the prescribed dietary regimen.
Luisi et al. (2019)	Hypocaloric Mediterranean diet Dietary intake measure: The adherence to the MD was evaluated by a score from 0 to 18 (0 minimal adherence; 18 greatest adherence). Diet components: Low-calorie Mediterranean diet (kcal 1,552 ± 160) utilised 40 g/die of HQ-EVOO for 3 months as the only cooking and dressing fat. 55–60% carbohydrates (mainly complex ones) 25–30% polyunsaturated and monounsaturated fats 15–20% proteins	Mediterranean diet Dietary intake measure: The adherence to the MD was evaluated by a score from 0 to 18 (0 minimal adherence; 18 greatest adherence) Normal weight controls followed a typical Mediterranean diet enriched with 40 g/die HQ-EVOO for three months. 55–60% carbohydrates, mainly complex ones 25–30% polyunsaturated and monounsaturated fats 15–20% proteins	Data not available
Thomazella et al. (2011)	Mediterranean diet Dietary intake measure: dietary intake questionnaires and validated adherence scores from 0-9 (9 indicating best adherence). Diet components: Unrefined cereals and products, fresh fruits (4 to 6 servings/day); varied raw or cooked vegetables and legumes (2 to 3 servings/day); extra-virgin olive oil (30 ml/day) as the main added fat; nonfat or low-fat dairy products (1–2 servings/day) and nuts (10 g/day); (2) weekly consumption of fish (3 to 4 times/week), poultry (3 to 4 times/week), and eggs (0 to 4 per week) and low red meat consumption (once a week). Sweets were allowed only a few times per month; red wine consumption (250 ml/day) was recommended for all MD patients. 12%-17% of total calories from protein 45%-50% of total calories from carbohydrate 33%-38% of total calories from fat	Low-fat Therapeutic Lifestyle Changes Diet Dietary intake measure: dietary intake questionnaires Diet components: Decreased fat intake, particularly saturated and trans-fatty acids; increased intake of fruits, vegetables, legumes, whole grains, fat-free and low-fat dairy products; moderate amount of lean meat, fish, or poultry; and vegetable oil for cooking. Daily consumption of soluble fibre-rich foods and all were asked to avoid alcohol during the study. Approximately 15% of total calories from protein 55%-60% of total calories from carbohydrate 25%-30% of total calories from fat	Validated adherence scores showed values of 7, 8, and 9, respectively, in 19%, 33%, and 48% of MD patients. Reasons for scores of 7 and 8 were fish intake <3 times/week and/or lower compliance with whole-grain cereals.

### Risk of bias assessment

Eligible studies were assessed to show risk of bias through criteria provided by the Academy of Nutrition and Dietetics (Table 6). The criteria provided questions on the clarity of research, selection bias, comparability of study groups, withdrawal handling, study protocol and statistical analysis. Questions are listed in Table 3 of the American Dietetic Association (ADA) Evidence Analysis Manual (Academy of Nutrition and Dietetics, 2016). If most of the answers to the validity questions were “Yes” (including criteria 2, 3, 6, 7 and at least one additional “Yes”), the report was given a plus symbol ( + ). If the answers to validity criteria questions 2, 3, 6, and 7 did not indicate that the study is exceptionally strong, the report was given a neutral (Æ) symbol. If most (six or more) of the answers to the validity questions were “No,” the report was given a minus (-) symbol. Table 6 presents the quality scores for the included studies. Regardless of validity rating, all studies included in quality analysis were included in the review.

**Table 6.** Quality assessment of included studies.

Author, year	1. Was the research question clearly stated?	2. Was the selection of study subjects/patience free from bias	3. Were study groups comparable	4. Was method of handling withdrawal described	5. Was blinding used to prevent introduction of bias	6. were protocols described?	7. Were outcomes clearly defined and the measurement valid and reliable?	8. Was the statistical analysis appropriate for the study design?	9. Are conclusions supported by results with biases and limitations considered?	10. Is bias due to study's funding or sponsorship unlikely?	TOTAL “Y”	Quality assessment score
Stendell-Hollis et al. (2013)	Y	Y	Y	Y	Y	Y	Y	Y	Y	Y	10	+
Marques-Rocha et al. (2016)	Y	Y	N/A	N	N	Y	Y	Y	Y	Y	7	+
Sofi et al. (2018)	Y	Y	Y	Y	N	Y	Y	Y	Y	Y	9	+
Perticone et al. (2019)	Y	Y	Y	Y	N	Y	Y	Y	Y	Y	9	+
Luisi et al. (2019)	Y	Y	Y	N	N	Y	Y	Y	Y	Y	8	+
Thomazella et al. (2011)	Y	Y	Y	Y	Y	Y	Y	Y	Y	Y	10	+

## Results

### Selection process

**Figure 1.**
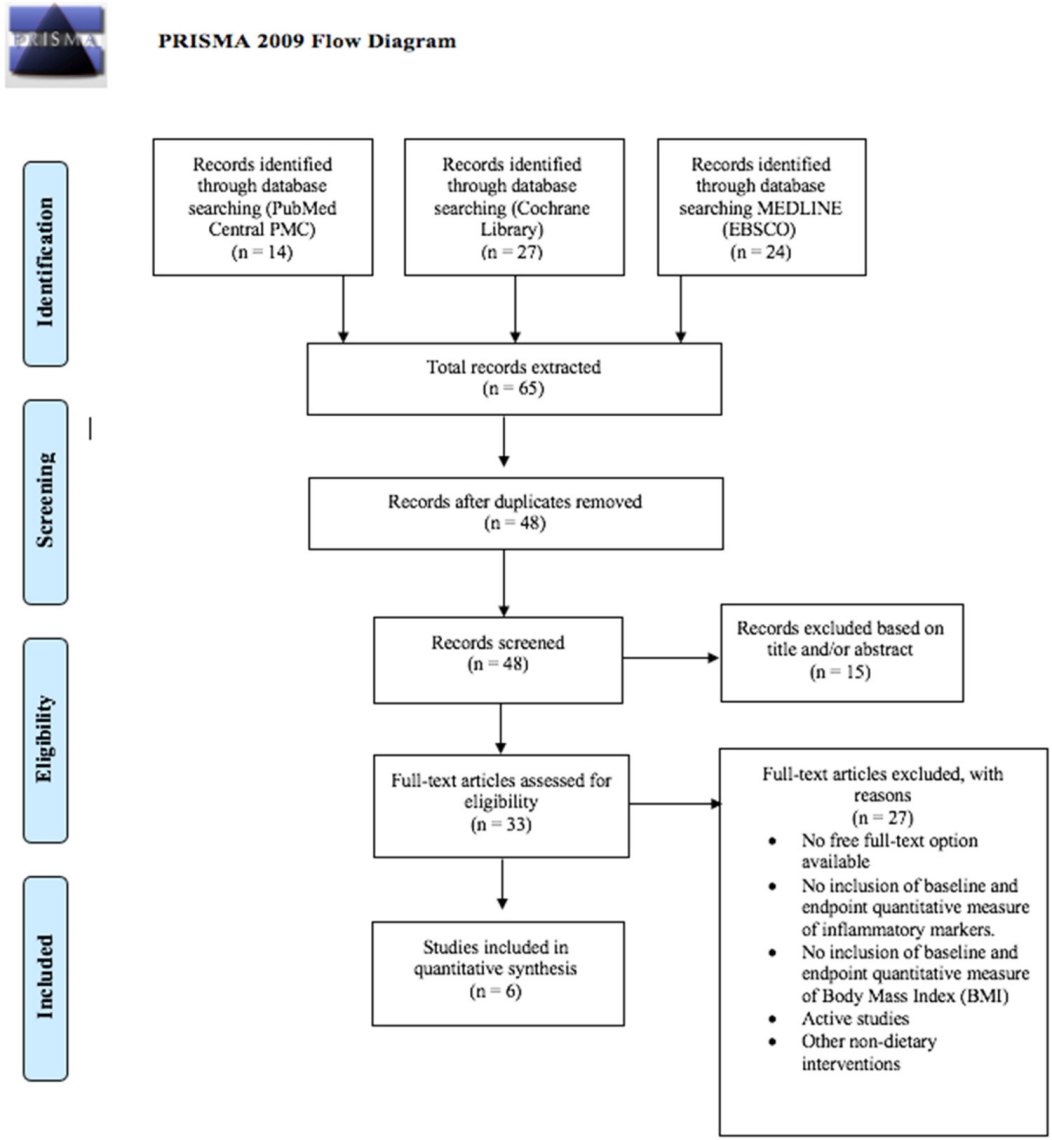
PRISMA flow diagram. The process of study identification, screening, inclusion and exclusion.

Figure 1 displays the PRISMA flow chart of the study selection process. The systematic database search located 65 studies, fourteen from PubMed, 27 from Cochrane Library and 24 from MEDLINE. Once duplicates had been removed, 48 studies remained eligible for screening, fifteen studies were then excluded based on their title and/or abstract, due to the lack of relevance to the inclusion criteria. A further 27 studies were excluded on the basis that one or more of the following applied: (1) full-text accessibility was not available (2) there was no inclusion of baseline and/or endpoint quantitative measures of BMI and/or inflammatory markers (3) the study included the implementation of other non-dietary interventions (e.g., physical activity programs) (4) the study was active. Out of 46 extracted articles, six (Luisi et al., 2019; Marques-Rocha et al., 2016; Perticone et al., 2019; Sofi et al., 2018; Stendell-Hollis et al., 2013; Thomazella et al., 2011) studies remained eligible for inclusion in the qualitative synthesis. Excluded studies are presented in S1.

### Study characteristics

Full study characteristics are presented in Table 3, including specific study aims, study design, participant demographic, eligibility criteria, follow up period, country/location. The risk of bias assessment for each study is presented in Table 6 and Table 5 provides a summary of diet interventions. Three studies were carried out in Italy (Luisi et al., 2019; Perticone et al., 2019; Sofi et al., 2018), one in the U.S.A (Stendell-Hollis et al., 2013), one in Spain (Marques-Rocha et al., 2016) and one in Brazil (Thomazella et al., 2011). Out of the six eligible studies, two included a standard MD intervention, four introduced a variation of the MD, four were hypocaloric MD and one enriched with 40 g/day High Quality-Extra Virgin Olive Oil. Luisi et al. (2019) was the only study (n = 36) that included a normal weight control group (BMI 18.5–24.9 kg/m^2^, n = 18) against overweight/obese participants (BMI ≥25 kg/m^2^, n = 18) of which a similar metabolic health profile was reported between both study groups, yet slightly increased amongst cases. The control group received a typical MD in the absence of calorie restriction, and cases received a low-calorie |MD (1552 ± 160 kcal/day).

Four studies involved two study arms with comparison interventions, including the MyPyramid (MP) diet for Pregnancy and Lactation (Stendell-Hollis et al., 2013), a low-calorie vegetarian diet (LCVD) (Sofi et al., 2018), a very low-calorie ketogenic diet (VLCKD) (Perticone et al., 2019) and a low-fat Therapeutic Lifestyle Changes Diet (TLCD) (Thomazella et al., 2011). Marques-Rocha et al. (2016) conducted an 8-week hypocaloric controlled intervention study, based on the reduction of metabolic syndrome in Navarra, Spain diet (RESMENA), with no inclusion of control groups or study arms. All participants taking part in a MD intervention had a mean baseline BMI of ≥25 kg/m^2^, varying from 26.5 kg/m^2^ to 38.8 kg/m^2^. The included studies used varying durations, from eight weeks (Marques-Rocha et al., 2016), 3 months (Luisi et al., 2019; Sofi et al., 2018; Thomazella et al., 2011), four months (Stendell-Hollis et al., 2013) to 12 months (Perticone et al., 2019).

### Mediterranean style diets

A complete summary of diet interventions and measures of dietary adherence is presented in Table 5. Two studies included a standard MD intervention, but both reported as hypocaloric by nature at follow up (Stendell-Hollis et al., 2013; Thomazella et al., 2011). In comparison, four (Luisi et al., 2019; Marques-Rocha et al., 2016; Perticone et al., 2019; Sofi et al., 2018) of the studies introduced a variation of the MD, all were hypocaloric and one (Luisi et al., 2019) enriched with 40 g/day High Quality-Extra Virgin Olive Oil. The similarities amongst all MD interventions included the consumption of whole grains, fruit and vegetables, fish, nuts, olive oil, legumes and poultry. As displayed in Table 5, macronutrient requirements varied, but remained in similar margins to each other. Overall, one study did not report any data on macronutrient intake (Stendell-Hollis et al., 2013). Generally, the range of energy derived from carbohydrate was 45% to 60%, protein was 10% to 24.6% and fat was 25% to 38% (Luisi et al., 2019; Marques-Rocha et al., 2016; Perticone et al., 2019; Sofi et al., 2018; Thomazella et al., 2011).

### Risk of bias assessment

The risk of bias assessment scores are presented in Table 6. All studies included in the current review received a positive ( + ) rating, indicating that the majority of validity questions were answered as “YES” out of maximum 10. In total, two studies scored 10/10 (Stendell-Hollis et al., 2013; Thomazella et al., 2011), two studies scored 9/10 (Perticone et al., 2019; Sofi et al., 2018), one scored 8/10 (Luisi et al., 2019) and one study scored 7/10 (Marques-Rocha et al., 2016). Marques-Rocha et al. (2016) received the lowest rating of 7 total “YES” due to the lack of blinding and limited description of subject withdrawal handling. However, question 3 was excluded due to lack of relevance, resulting in the lowest score.

### Effects of the Mediterranean diet on BMI

Out of the six eligible studies, reductions in BMI measurement at follow up were consistent after consuming a MD, five of which were significant (Table 4). Significant reductions in BMI (P < 0.05) were reported amongst all participants whose mean BMI fell within the range of 30.2 kg/m^2^ to 35.4 kg/m^2^, however a non-significant reduction was reported for participants with a mean BMI of 38.8 kg/m^2^. All overweight participants, with mean BMI ranging from 26.5 kg/m^2^ to 27.1 kg/m^2^, reported significant reductions (P < 0.001). Stendell-Hollis et al. (2013) showed reductions in mean BMI to be significant (P < 0.001) at 4 months follow up (27.1 ± 5.29 to 26.2 ± 5.58 ( − 0.85 ± 1.24)) as well as in the comparative MP diet group (26.7 ± 4.82 to 25.6 ± 5.24 ( − 1.13 ± 1.22)). Likewise, Marques-Rocha et al. (2016) showed a significant reduction (P < 0.01) in BMI across participants who consumed the calorie restricted RESMENA diet at the 8 weeks follow up (35.4 ± 4.4 to 32.8 ± 4.2). When compared with a hypocaloric MD, Sofi et al. (2018) reported greater reductions in BMI in the LCVD group ( − 0.67kg/m^2^ and − 0.64kg/m^2^) but both interventions generated significant reductions for BMI for obese participants (P < 0.05). Likewise, Luisi et al. (2019) reported significant reductions in mean BMI for overweight/obese controls (P < 0.01) at the three months follow up (30.2 ± 1.0 to 28.8 ± 0.9) and remained statistically significant (P < 0.05) when compared to normal weight controls (21.6 ± 0.6 to 21.7 ± 0.6). Thomazella et al. (2011) reported similar significant reductions (<0.001) in BMI measurement in both intervention groups at three months follow up (MD (26.5 ± 1.9 to 25.9 ± 1.8) and TLCD (26.3 ± 2.5 to 25.7 ± 2.4). In contrast, Perticone et al. (2019) showed no significant reductions in BMI measurement from the standard hypocaloric MD (38.8 ± 4.5 to 36.1 ± 5.7).

### Effects of the Mediterranean diet on inflammatory markers

The baseline and endpoint inflammatory markers are presented in Table 4. Consumption of a MD showed reductions in hs-CRP in two studies. Perticone et al. (2019) showed significant reductions (P = 0.044) in hs-CRP (mg/L) after twelve months in participants who consumed the standard hypocaloric MD (5.6 ± 4.3 to 3.7 ± 1.2) as well as significant reductions (P < 0.0001) in the VLCKD (4.5 ± 2.6 to 1.8 ± 0.8). Thomazella et al. (2011) showed reductions in mean hs-CRP after three months in the MD group (1.65 ± 1.50 to 1.07 ± 0.93) but changes were not significant. MD consumption offered no significant changes in CRP for the included studies

Stendell-Hollis et al. (2013) reported a significant reduction (P = 0.021) in TNF-α (pg/mL) in the MD group (4.189 pg/mL (1.30–7.08) to 3.301 (1.10–5.50)) and significant reductions (P < 0.001) in the MP diet group (2.475 pg/mL (1.48–3.47) to 1.940 (1.18–2.70)). Likewise, Luisi et al. (2019) showed significant reductions (P < 0.001) in both overweight/obese participants (1.5 to 1.1 pg/mL) who consumed a low-calorie MD. Sofi et al. (2018) showed non-significant reductions in mean TNF-α for the low calorie MD group after three months (3.20 pg/mL (2.53–4.04 to 2.86 pg/mL) (2.12–3.87). In contrast, Marques-Rocha et al. (2016) showed non-significant increases in TNF-α (pg/mL) after eight weeks.

Measure of interleukins included IL-1ra, IL-4, IL-6, IL-8, IL-10, IL-12 and IL-17. Two studies reported findings on Interleukin-10 (IL-10) (pg/mL). Luisi et al. (2019) reported increases in IL-10 in both the overweight/obese participants (5.5 to 7.5) who consumed a low-calorie MD, and normal weight controls (7 to 7.5) who consumed a typical MD. However, only increases in the overweight/obese participants were reported as significant (P < 0.001). Sofi et al. (2018) showed a reduction in IL-10 (1.81 (1.37–2.37) to 1.50 (1.14–1.95)), IL-12 (16.48 (14.11–19.26) to 14.35 (12.45–16.59); P < 0.05) and IL-1ra (13.45 (11.43–15.82) to 10.70 (9.23–12.39); P < 0.05) for participants consuming a low-calorie MD.

Three of the four studies that measured Interleukin-6 (IL-6) reported reductions, one of which was significant. Luisi et al. (2019) reported similar significant decreases in IL-6 in both normal weight controls (4 ng/mL to 3.2 ng/mL; p < 0.001) who consumed a typical MD, and overweight/obese participants (4.67 ng/mL to 3.87 ng/mL; p < 0.001) who consumed a low-calorie MD. None of the included studies reported significant reductions in IL-8.

Marques-Rocha et al. (2016) reported significant reductions (P < 0.01) in mean PAI-1 (pg/mL) levels (157 ± 127 to 144 ± 152) and significant reductions (P < 0.01) in mean MDA (mM) levels after 8 weeks of the low-calorie RESMENA diet (0.84 ± 0.36 to 0.74 ± 0.28).

No significant increases were shown in IFN-γ levels amongst eligible studies at follow up after a MD intervention. Sofi et al. (2018) showed significant reductions (P < 0.05) in MCP-1 (pg/mL) (P < 0.05) and MIP-1β (pg/mL) at three month follow up in participants who consumed the low-calorie MD (22.76 (20.05–25.87) to 17.98 (16.17–19.97) and (52.40 (47.66–57.57) to 45.47 (41.06–50.40) respectively). Sofi et al. (2018) also showed significant reductions (P < 0.05) in mean VEGF (42.86 (35.80–51.32) to 36.16 (30.51–42.91) (pg/mL) and IP-10 (pg/mL) (475.33 (427.95–527.95) to 447.20 (407.48–490.78)) levels at three months follow up in participants who consumed the low-calorie MD.

## Discussion

### Effects of Mediterranean diet on BMI

This systematic review aimed to investigate the effects of consuming a MD on the BMI of overweight/obese adults to ascertain its potential as a COVID-19 mitigation strategy. BMI reductions amongst MD groups appear similar, to those from comparative dietary interventions amongst overweight/obese adults. Results confirm reductions in mean BMI post- MD intervention, including studies without intentional calorie restriction. As reported by Thomazella et al. (2011) mean calorie intake in the standard MD group showed a total reduction of −464 kcal/day at three months follow up, which likely to contributed to the significant BMI reduction of −0.6 kg/m^2^ (Mancini et al., 2016). Similar calorie reductions were reported in the TLCD group (−478 kcal/day) with identical reductions in BMI (–0.6 kg/m^2^). With similarities between diets, the interventions differentiate by placing emphasis on overall fat intake reduction, alcohol avoidance and daily consumption of soluble fibre-rich foods in the TLCD, allowing lower calorie intake via restriction and satiation from fibre-rich foods (Warrilow et al., 2019). Conclusions support findings from D’Innocenzo et al. (2019) whereby adherence to a fibre-dense, MD based on plant-based, energy-low foods has more favourable effects on reducing BMI as opposed to a western diet that is higher in saturated fat, alcohol consumption and lower in fruit and vegetable consumption (Kopp, 2019).

Similar results were shown by Stendell-Hollis et al. (2013), whereby overweight, postpartum/breastfeeding women who consumed a standard MD showed significant decreases in calorie intake after four months ( − 251.2 kcal/day, p = 0.045), this was also reported in the MP group ( − 437.5 kcal/day; P = 0.035). It is possible that the deemphasis on fat intake, specifically from nuts and olive oil, could be causal for these greater calorie reductions in the MP group, which was introduced to differentiate the MP diet from the MD. The reduction in fat intake also provides a likely explanation for greater loss in BMI (−1.1 kg/m^2^) compared to the MD group (−0.9 kg/m^2^). However, the added energy requirements of lactation could account for the BMI reductions, whereby women who breastfeed can require 500 additional kcal/day beyond what is recommended for non-pregnant women (Kominiarek and Rajan, 2016). Therefore, increased energy demands throughout lactation may have aided towards the total reductions seen similarly in each study group, whereby a total 0.5–1.0 kg/month can be lost after the first postpartum month (Kominiarek and Rajan, 2016).

Studies that intervened with a hypocaloric/low-calorie MD diet all showed BMI reductions, majority significant, amongst overweight and obese adults. Sofi et al. (2018) reported similar reductions in BMI in the low-calorie MD group ( − 0.64 kg/m^2^) and the LCVD group ( − 0.67 kg/m^2^) amongst obese adults. Despite greater reductions in the LCVD group, it is indicated that close reductions between groups may be related to higher intakes of fibre-dense food groups such as complex carbohydrates, legumes, fruits, and vegetables, aiding weight loss via satiety, fat reduction and glucose absorption (Lattimer and Haub, 2010; Sofi et al., 2018). Although the intervention diets did not differ in the percentage of calories obtained from macronutrients and the main categories of food, the LCVD group were restricted from consuming any animal products. Therefore, the possibility of a greater reduction in calorie consumption in the LCVD cannot be excluded. Despite similar reductions, the total absence of these food items combined with a substitutional increase in fibre-dense foods, from vegetables and wholegrains, in the LCVD would have likely caused a greater caloric deficit in comparison to the MD group, likely accounting for greater BMI reduction (Najjar and Feresin, 2019).

This is further supported by Luisi et al. (2019) who reported significant reductions amongst overweight/obese participants intervened with a low-calorie MD for 3 months. In comparison to normal weight controls (BMI 18.5–24.9 kg/m^2^), participants restricted their caloric intake to 1552 ± 160 kcal/day. Despite duplicate macronutrient requirements reductions in BMI were only reported amongst overweight/obese participants after three months, whereby mean BMI amongst controls had a non-significant increase of + 0.1 kg/m^2^. These findings support that a hypocaloric |MD (1552 ± 160 kcal/day) can provide favourable, short-term impacts on BMI amongst overweight and/or obese adults. However, it has been shown that significant BMI reductions may yield from even shorter intervention durations (>8 weeks) as concluded by Marques-Rocha et al. (2016) whereby obese participants achieved a BMI loss of −2.6 kg/m^2^of after only 8-weeks of a hypocaloric MD intervention.

Similar conclusions can be made from Perticone et al. (2019). Despite no significance, greater reductions in mean BMI were reported in the VLCKD group in comparison to the standard hypocaloric Mediterranean diet (SHMD) at 12 months follow-up. Caloric requirements between intervention groups varied, whereby obese participants in the VLCKD consumed no greater than 800 kcal/day for 12 months. The VLCKD supports the reduction of total intake of both carbohydrates and lipids, whereby a calorie restriction of <800 kcal per day has been used as an effective regimen for weight loss. The ketone bodies produced in a ketogenic state are utilised to suppress appetite, allowing a minimal calorie intake (Kelly et al., 2020; Perticone et al., 2019). Participants in the VLCKD were also recommended 0.8–1.5 g of protein/kg of adequate body weight, allowing the satiation effects to yield greater potential weight loss and improve adherence. Comparatively, participants in the SHMD maintained a 500 kcal/day caloric deficit, whereby macronutrient advisories compared to those in the VLCKD, allowing greater amounts of energy to be derived from carbohydrates and fats, and less from proteins whereby adherence was much lower than stated in the VLCKD. These findings challenge conclusions made by Mancini et al. (2016) surrounding the hypothesis that there are “no optimal macronutrient composition for achieving sustained weight loss”, whereby macronutrient variations that advocate ketosis could optimise long-term weight reductions. Despite the likelihood of greater BMI reduction to be a product of heterogeneity surrounding calorie intake, sustained weight loss through ketogenic macronutrient variations in parallel with MD principles could surround potential future research for longer-term (>4 month) obesity mitigation (Mancini et al., 2016).

### Effects of Mediterranean diet on inflammatory markers

The current review aimed to investigate the effects of consuming a MD on the inflammatory markers of overweight/obese adults, whereby the inclusion of plant-derived nutritional components remain integral in providing bioactive compounds. These compounds present health-promoting effects due to their anti-inflammatory properties, whereby exaggerated inflammatory response has been linked with COVID-19 severe illness (Angelidi et al., 2021). Findings from the current review remain in support of this, whereby inflammatory markers seen elevated in COVID-19 severity, have been significantly reduced after dietary interventions surrounding MD constituents.

Results demonstrate that TNF-α, seen elevated in severe COVID-19 cases, have been significantly reduced (P = 0.021; P < 0.001) over a 3–4-month MD intervention for overweight/obese adults (Luisi et al., 2019; Stendell-Hollis et al., 2013). Findings from Stendell-Hollis et al. (2013) highlight the inflammatory-modulating effect of short-term MD and MP diets (4 months) on overweight adults, whereby participants in both groups showed significant reductions (P = 0.021). The deemphasis on fat intake, specifically from nuts and olive oil, was placed on the MP group to differentiate the MP diet from the MD. However, there remained similarities amongst diets including predominant intakes of foods containing; alpha-linolenic acid, monounsaturated and polyunsaturated fatty acids, fibre, and antioxidants which support findings of potential inflammatory modifying effects resultant from MD dietary constituents (Ajani et al., 2004; Mori et al., 2003; Paschos et al., 2004; Zhao et al., 2004). It is possible that the lesser significance in TNF-α reductions reported in the MD group could be resultant from the lack of adherence to the dietary behavioural targets reported and could therefore add to the absence of statistically significant differences in inflammation between diet groups (Stendell-Hollis et al., 2013). Supporting these conclusions, Luisi et al. (2019) reported the likely cause of significant reduction in inflammatory markers, TNF-α and IL-6, after MD interventions was the protective role provided by polyphenols when supplementing with 40g of high-quality extra virgin olive oil (HQ-EVOO) per day, of which benefits have been strongly depicted in previous studies (Carpi et al., 2019; Yarla et al., 2018). Similar significant reductions in these inflammatory markers were also reported amongst the normal weight controls who received a duplicate supplementation of HQ-EVOO, further supporting its dietary protective roles in modifying inflammation for future interventions amongst those at risk of severe COVID-19.

In support of these findings, Sofi et al. (2018) also reported reductions in TNF-α in the low calorie Mediterranean diet (LCMD) group in contrast to non-significant increases in the LCVD group. In support of Al-Daghri et al. (2016) associations have been shown between circulating vitamin B_12_ concentrations with inflammatory cytokines such as TNF-α in adults, highlighting a potential association between vitamin B_12_ deficiency with elevated TNF-α levels (Miller, 2002). It is likely that a vegetarian diet, that often excludes sources of vitamin B_12_ (Woo et al., 2014), may heighten the risk of elevated TNF-α in this circumstance. However, it can be shown that short-term (>4 months) significant reductions in TNF-α are achievable in adherence to a MD that incorporates high polyphenol foods such as HQ-EVOO. HQ-EVOO, which is one of the fundamental foods of MD, is rich in monounsaturated oleic acid which poses impressive anti-inflammatory properties (Medeiros-de-Moraes et al., 2018). In light of the COVID-19 pandemic, further research is needed to evaluate the effects of vitamin B_12_ supplementation in adherence to vegetarian/vegan diets and its potential to maximise inflammatory reduction in overweight/obese adults.

Regarding hs-CRP, our findings highlight short-term (3 months) and longer-term (12 months) reductions in hs-CRP levels amongst overweight/obese adults who consumed a MD. Perticone et al. (2019) reported significant reductions in hs-CRP after twelve months in SHMD and VLCKD intervention groups, however reductions were greater and more significant in the VLCKD. Due to the associated correlations with BMI reduction, findings add to those reported by Merra et al. (2017) whereby adherence to a short term (3-weeks) very low-calorie ketogenic diet (450–700 kcal/day) remained parallel to a reduction in BMI (p = 0.001) and the inflammatory marker, CRP (p = 0.02) amongst adults with a BMI of ≥25 kg/m^2^, confirming the potential effectiveness of a VLCKD for risk markers of COVID-19 severity.

Regarding IL-6, short-term (3–4 months) reductions were reported amongst participants who consumed a MD, however, increases in IL-6 were reported amongst those who consumed a LCVD. Both study groups achieved significant reductions in BMI, however the unparallel link between IL-6 and BMI in the LCVD raises questions for recent conclusions devised by Jaceldo-Siegl et al. (2018), whereby lower IL-6 concentrations among vegetarians remained associated with BMI amongst overweight adult participants. Therefore, it may be concluded that a longer intervention is required to assess the association between a LCVD diet and IL-6, or findings could instead add to those from Samavat et al. (2018) whereby low vitamin B_12_ conditions, commonly presented in the presence of vegetarian diets, have aligned significantly with increased expression of pro-inflammatory cytokines such as interleukin-6 (IL-6) (Sofi et al., 2018). Although not significant, Stendell-Hollis et al. (2013) reported reductions in IL-6 amongst those who consumed the MD and MP diet at 4-months follow up. Participants in both groups also achieved significant reductions in BMI, which in turn would reflect recent findings from El-Mikkawy et al. (2020) whereby circulating level of IL-6 was associated with the intensity of the chronic and systemic inflammation that develops with high degrees of obesity amongst an adult population.

### Study strengths and limitations

Strengths are in line with the current review. The conclusions drawn from findings offer plausibility and importance surrounding potential dietary interventions for measures that are parallel to COVID-19 severity. The eligibility formation and data collection allow focus to be placed on the determinants of COVID-19 outcomes, with intent to deduct bias and create a strong foreground for future research into potential disease severity mitigation, through BMI reduction and modification of inflammatory markers. Limitations surrounding the current study should however also be noted. The inclusion of post-natal/breast feeding participants raises issues surrounding generalisability and bias. When analysing the study findings, it is important to be vigilant of the potential heterogeneity in outcome data resultant from the post-natal/breast feeding participant markers included, as underlying factors may cause discrepancies in interpretations. In addition, although the included studies adhere to participant eligibility and intervention outcomes, the limited attribution of randomisation amongst studies could create significant impediment through application of findings. Three out of six studies included randomisation in the intervention method, indicating that the elimination of bias amongst studies remains inconsistent and should be considered when analysing study findings. It is also essential to consider that short, 8-week intervention periods may enable misinterpretations in the potential MD intervention effectiveness. Consistent, long-term MD adherence studies in “real life” settings are required to assess the potential alterations in BMI and inflammatory markers needed for alterations in current dietary guidelines. In addition, concluded predictions from measured outcomes focus on BMI alterations and inflammatory modification resultant from current literature surrounding COVID-19, as opposed to direct mitigation of severity in COVID-19 patients. One study also did not report data on dietary adherence enabling misinterpretations of low-calorie MD intervention effectiveness for overweight and obese adults (Luisi et al., 2019). It is therefore essential to highlight the necessity for further research into various dietary patterns in line with the direct severity measures of COVID-19. A further systematic review/meta-analysis of COVID severity outcomes and dietary patterns should be considered as emerging evidence continues to become available in the future (Kim et al., 2021).

## Conclusions

To conclude, the results from this systematic review indicate that a hypocaloric, fibre-dense MD is an effective, short term (<4 months) mitigation strategy to significantly reduce BMI amongst overweight/obese adults who may be at a heightened risk of developing severe COVID-19 outcomes. High intakes of bioactive compounds, in line with MD adherence, have proven integral for their inflammation reducing benefits reported amongst overweight/obese adults, whereby the inclusion of HQ-EVOO alongside BMI reduction can maximise short-term potential. Our findings show a hypocaloric, fibre and protein dense MD that adheres towards restricted alcohol and saturated fat intake could provide sustained reductions in BMI (>4 months). Following ketogenic macronutrient recommendations (55% to 60% fat, 30% to 35% protein, and 5% to 10% carbohydrates) may further reinforce BMI reduction potential via appetite suppression and satiety amongst overweight/obese adults (Masood et al., 2021). The inclusion of polyphenol-dense foods, such as HQ-EVOO, in combination with a hypocaloric MD may also present favourable outcomes on inflammatory markers. These approaches could therefore be considered for future research into longer term interventions to tackle obesity and inflammatory regulation to help mitigate the ongoing COVID-19 pandemic.

## Supplemental Material

sj-docx-1-nah-10.1177_02601060221127853 - Supplemental material for The effects of consuming a Mediterranean style diet on associated COVID-19 severity biomarkers in obese/overweight adults: A systematic review Click here for additional data file. Supplemental material, sj-docx-1-nah-10.1177_02601060221127853 for The effects of consuming a Mediterranean style diet on associated COVID-19 severity biomarkers in obese/overweight adults: A systematic review by Ella Moore, Abdulmannan Fadel and Katie E. Lane in Nutrition and Health
