# Supplementary material for: The effects of consuming a Mediterranean style diet on associated COVID-19 severity biomarkers in obese/overweight adults: A systematic review
Source: Nutr Health. 2022 Sep 21;28(4):647–67. doi: 10.1177/02601060221127853 (PMC9494166; doi:10.1177/02601060221127853)
Supplement: sj-docx-1-nah-10.1177_02601060221127853 - Supplemental material for The effects of consuming a Mediterranean style diet on associated COVID-19 severity biomarkers in obese/overweight adults: A systematic review [file sj-docx-1-nah-10.1177_02601060221127853.docx]

**S1 - Supplementary Materials.**

References of excluded studies

Abete, I., J, Konieczna., M.A, Zulet., A.M, Galmés-Panades., I, Ibero-Baraibar., N, Babio., R, Estruch., J, Vidal., E, Toledo., C, Razquin., et al. 2019. Association of lifestyle factors and inflammation with sarcopenic obesity: data from the PREDIMED-Plus trial. *Journal of cachexia, sarcopenia and muscle* 10 (5): 974–984. doi: 10.1002/jcsm.12442

Baguley, B.J., T.L, Skinner., D.G, Jenkins., O.R.L, Wright. 2020. Mediterranean-style dietary pattern improves cancer-related fatigue and quality of life in men with prostate cancer treated with androgen deprivation therapy: A pilot randomised control trial. *Clin Nutr* 40 (1): 245-254. DOI: 10.1016/j.clnu.2020.05.016

Barrea, L., A, Arnone., G, Annunziata., G, Muscogiuri., D, Laudisio., C, Salzano., G, Pugliese., A, Colao., S, Savastano. 2019. Adherence to the Mediterranean Diet, Dietary Patterns and Body Composition in Women with Polycystic Ovary Syndrome (PCOS). *Nutrients* 11 (10): 2278. doi: 10.3390/nu11102278

Barrea, L., G, Muscogiuri., D, Laudisio., G, Pugliese., G, de Alteriis., A, Colao., S, Savastano. 2020. Influence of the Mediterranean Diet on 25- Hydroxyvitamin D Levels in Adults. *Nutrients* 12 (5): 1439. doi: 10.3390/nu12051439

Capurso, C., G, Vendemiale. 2017. The Mediterranean Diet Reduces the Risk and Mortality of the Prostate Cancer: A Narrative Review. *Frontiers in nutrition* 4: 38. doi: 10.3389/fnut.2017.00038

Damasceno, N.R., A, Pérez-Heras., M, Serra., M, Cofán., A, Sala-Vila., J, Salas-Salvadó., E, Ros. 2011. Crossover study of diets enriched with virgin olive oil, walnuts or almonds. Effects on lipids and other cardiovascular risk markers. *Nutr Metab Cardiovasc Dis* 21 (1): S14-20. doi: 10.1016/j.numecd.2010.12.006

Diekmann, C., H, Huber., M, Preuß., P, Preuß., H.G, Predel., B, Stoffel-Wagner., R, Fimmers., P, Stehle., S, Egert. 2019. Moderate Postmeal Walking Has No Beneficial Effects Over Resting on Postprandial Lipemia, Glycemia, Insulinemia, and Selected Oxidative and Inflammatory Parameters in Older Adults with a Cardiovascular Disease Risk Phenotype: A Randomized Crossover Trial. *J Nutr* 149 (11): 1930-1941. doi: 10.1093/jn/nxz148.

Giroli, M.G., M, Amato., V, Cavalca., J.P, Werba., A, Di Minno., B, Porro., A, Bonomi., L, Vigo., E, Tremoli., F, Veglia. 2019. Effect of mediterranean diet on inflammatory markers and classical risk factors in patients with coronary heart disease. *European journal of preventive cardiology* 26 (8): S136‐. https://doi.org/10.1177/2047487319860056

Gomez-Huelgas, R., J, Ruiz-Nava., S, Santamaria-Fernandez., A, Vargas-Candela., A.V, Alarcon-Martin., F.J, Tinahones., M.R, Bernal-Lopez. 2019. Impact of Intensive Lifestyle Modification on Levels of Adipokines and Inflammatory Biomarkers in Metabolically Healthy Obese Women. *Mediators of inflammation* 2019: 4165260. doi: 10.1155/2019/4165260

Hill, E., A, Hodge., P, Clifton., N, Shivappa., J.R, Hebert., L, Dennerstein., S, Campbell., C, Szoeke. 2019. Longitudinal nutritional changes in aging Australian women. *Asia Pac J Clin Nutr* 28 (1): 139-149. doi: 10.6133/apjcn.201903_28(1).0019.

Kenđel Jovanović, G., S, Pavičić Žeželj., S, Klobučar MajanovićS., I, Mrakovcic-Sutic., I, Šutić. 2019. Metabolic syndrome and its association with the Dietary Inflammatory Index (DII)® in a Croatian working population*. J Hum Nutr Diet* 33 (1): 128-137. doi: 10.1111/jhn.12695

Kolehmainen, M., S, Ulven., J, Paananen., V, de Mello., U, Schwab., C, Carlberg., M, Myhrstad., J, Pihlajamäki., E, Dungner., E, Sjölin., et al. 2014. Healthy Nordic diet downregulates the expression of genes involved in inflammation in subcutaneous adipose tissue in individuals with features of the metabolic syndrome. *The American Journal of Clinical Nutrition* 101 (1): 228-239. doi: 10.3945/ajcn.114.092783

Kontogianni, M.D., N, Tileli., A, Margariti., M, Georgoulis., M, Deutsch., D, Tiniakos., E, Fragopoulou., R, Zafiropoulou., Y, Manios., G, Papatheodoridis. 2014. Adherence to the Mediterranean diet is associated with the severity of non-alcoholic fatty liver disease. *Clin Nutr* 33 (4): 678-83. doi: 10.1016/j.clnu.2013.08.014.

Kouvari, M., D.B, Panagiotakos., M, Yannakoulia., E, Georgousopoulou., E, Critselis., C, Chrysohoou., D, Tousoulis., C, Pitsavos. 2019. Transition from metabolically benign to metabolically unhealthy obesity and 10-year cardiovascular disease incidence: The ATTICA cohort study. *Metabolism* 93: 18-24. doi: 10.1016/j.metabol.2019.01.003

Kyrou, I., D.B, Panagiotakos., G.M, Kouli., E, Georgousopoulou., C, Chrysohoou., C, Tsigos., D, Tousoulis., C, Pitsavos. 2018. Lipid accumulation product in relation to 10-year cardiovascular disease incidence in Caucasian adults: The ATTICA study. *Atherosclerosis* 279: 10-16. doi: 10.1016/j.atherosclerosis.2018.10.015.

Mancioppi, V., A, Solito., R, Ricotti., D, Carrera., F, Archero., V, Landoni., R, Errichiello., E, Bona., M, Cavaletto., S, Bellone., et al. 2019. Good-day: efficacy of gamification of an educational training to mediterranean diet on weight and metabolic control in paediatric obesity. preliminary data at 6 months. *High blood pressure and cardiovascular prevention* 26 (2):171‐172. DOI: https://doi.org/10.1007/s40292-019-00308-6

Mohorko, N., Z, Jenko-Pražnika., A, Petelin. 2016. Leucine and lysine intakes are highly associated with serum adiponectin levels in asymptomatic adults. *Minerva Endocrinol* 41 (3): 302-13. Available at: <https://pubmed.ncbi.nlm.nih.gov/25677829/>

Monfort-Pires, M., S.R, Ferreira. 2017. Inflammatory and metabolic responses to dietary intervention differ among individuals at distinct cardiometabolic risk levels. *Nutrition* 33: 331-337. doi: 10.1016/j.nut.2016.07.021

Mucci, F., D, Marazziti., A, Della Vecchia., S, Baroni., G, Massimetti., P, Morana., P, Mangiapane., F, Morana., B, Carpita., B, Morana., et al. 2020. Inflammatory and metabolic markers in patients with mood disorders. *World J Biol Psychiatry* 18: 1-8. doi: 10.1080/15622975.2020.1775891.

Nordmann, A.J., K, Suter-Zimmermann., H.C, Bucher., I, Shai., K.R, Tuttle., R, Estruch., M, Briel. 2011. Meta-analysis comparing Mediterranean to low-fat diets for modification of cardiovascular risk factors. *Am J Med* 124 (9): 841-51. DOI: 10.1016/j.amjmed.2011.04.024

Park, Y.M., J, Zhang., S.E, Steck., T.T, Fung., L.J, Hazlett., K, Han., S.H, Ko., A.T, Merchant. 2017. Obesity Mediates the Association between Mediterranean Diet Consumption and Insulin Resistance and Inflammation in US Adults. *The Journal of nutrition* 147 (4): 563–571. doi: 10.3945/jn.116.243543

Parlapani, E., C, Agakidis., T, Karagiozoglou-Lampoudi., K, Sarafidis., E, Agakidou., A, Athanasiadis., E, Diamanti. 2019. The Mediterranean diet adherence by pregnant women delivering prematurely: association with size at birth and complications of prematurity. *J Matern Fetal Neonatal Med* 32 (7): 1084-1091. doi: 10.1080/14767058.2017.1399120

Parsons, T. J., E, Papachristou., J.L, Atkins., O, Papacosta., S, Ash., L.T, Lennon., P.H, Whincup., S.E, Ramsay., S.G, Wannamethee. 2019. Physical frailty in older men: prospective associations with diet quality and patterns. *Age and ageing* 48 (3): 355–360. doi: 10.1093/ageing/afy216

Ramirez, A.G., D.L, Parma., E, Muñoz., K.D, Mendoza., C, Harb., A, Holden., M, Wargovich. 2017. An anti-inflammatory dietary intervention to reduce breast cancer recurrence risk: Study design and baseline data. *Contemporary clinical trials* 57: 1–7. doi: 10.1016/j.cct.2017.03.009

Ruiz-Canela, M., I, Zazpe., N, Shivappa., J.R, Hébert., A, Sánchez-Tainta., D, Corella., J, Salas-Salvadó., M, Fitó., R.M, Lamuela-Raventós., J, Rekondo., et al. 2015. Dietary inflammatory index and anthropometric measures of obesity in a population sample at high cardiovascular risk from the PREDIMED (PREvención con DIeta MEDiterránea) trial. *The British journal of nutrition* 113 (6): 984–995. DOI: doi: 10.1017/S0007114514004401

Salas‐Salvado, J. 2015. Nuts and cardiometabolic health. *Annals of Nutrition and Metabolism* 67 (1): 1-601. DOI: https://doi.org/10.1159/000440895

Salas-Salvadó, J., M, Guasch-Ferré., M, Bulló., J, Sabaté. 2014. Nuts in the prevention and treatment of metabolic syndrome. *Am J Clin Nutr* 100 (1): 399S-407S. doi: 10.3945/ajcn.113.071530

Stradling, C., S, Taylor., G.N, Thomas., S, Taheri., J, Ross., S, Das. 2017. Mediterranean diet can improve cardiovascular risk in HIV dyslipidaemia: a randomised controlled dietary intervention trial. *HIV medicine* 18: 41-. DOI: https://doi.org/10.1111/hiv.12513

Tantucci, A., A, Timi., G, Perriello. 2018. Influence of dietary and haematobiochemical profile in patients with type 2 diabetes belong to Umbria clinic: “TOSCA.IT” study. *Diabetologia* 61: S339‐ DOI: https://doi.org/10.1007/s00125-018-4693-0

Tussing-Humphreys, L., M, Lamar., J.A, Blumenthal., M, Babyak., G, Fantuzzi., L, Blumstein., L, Schiffer., M.L, Fitzgibbon. 2017. Building research in diet and cognition: The BRIDGE randomized controlled trial. *Contemporary clinical trials* 59: 87–97. doi: 10.1016/j.cct.2017.06.003

Wade, A. T., C.R, Davis., K.A, Dyer., J.M, Hodgson., R.J, Woodman., H.A, Keage., K.J, Murphy. 2017. A Mediterranean Diet to Improve Cardiovascular and Cognitive Health: Protocol for a Randomised Controlled Intervention Study. *Nutrients* 9 (2): 145. doi: 10.3390/nu9020145

Yubero-Serrano, E.M., L, Gonzalez-Guardia., O, Rangel-Zuñiga., J, Delgado-Lista., F.M, Gutierrez-Mariscal., P, Perez-Martinez., N, Delgado-Casado., C, Cruz-Teno., F.J, Tinahones., J.M, Villalba., et al. 2012. Mediterranean diet supplemented with coenzyme Q10 modifies the expression of proinflammatory and endoplasmic reticulum stress-related genes in elderly men and women. *J Gerontol A Biol Sci Med Sci* 67 (1): 3-10. DOI: doi: 10.1093/gerona/glr167
